# Expression Profiles of 2 Phosphate Starvation-Inducible Phosphocholine/Phosphoethanolamine Phosphatases, PECP1 and PS2, in Arabidopsis

**DOI:** 10.3389/fpls.2019.00662

**Published:** 2019-05-29

**Authors:** Artik Elisa Angkawijaya, Anh H. Ngo, Van C. Nguyen, Farrel Gunawan, Yuki Nakamura

**Affiliations:** ^1^Institute of Plant and Microbial Biology, Academia Sinica, Taipei, Taiwan; ^2^Molecular and Biological Agricultural Sciences Program, Taiwan International Graduate Program, Taipei, Taiwan; ^3^Graduate Institute of Biotechnology, National Chung Hsing University, Taichung, Taiwan; ^4^Biotechnology Center, National Chung Hsing University, Taichung, Taiwan

**Keywords:** phosphate starvation, membrane lipid remodeling, phospholipids, phosphatase, *Arabidopsis thaliana*

## Abstract

Phosphorus is essential for plant viability. Phosphate-starved plants trigger membrane lipid remodeling to replace membrane phospholipids by non-phosphorus galactolipids presumably to acquire scarce phosphate source. Phosphoethanolamine/phosphocholine phosphatase 1 (PECP1) and phosphate starvation-induced gene 2 (PS2) belong to an emerging class of phosphatase induced by phosphate starvation and dephosphorylates phosphocholine and phosphoethanolamine (PEtn) *in vivo*. However, detailed spatiotemporal expression pattern as well as subcellular localization has not been investigated yet. Here, by constructing transgenic plants harboring functional translational promoter–reporter fusion system, we showed the expression pattern of PECP1 and PS2 in different tissues and in response to phosphate starvation. Besides, the Venus fluorescent reporter revealed that both are localized at the ER. Characterization of transgenic plants that overexpress PECP1 or PS2 showed that their activity toward PEtn may be different *in vivo*. We suggest that PECP1 and PS2 are ER-localized phosphatases that show similar expression pattern yet have a distinct substrate specificity *in vivo.*

## Introduction

**FIGURE 1:**
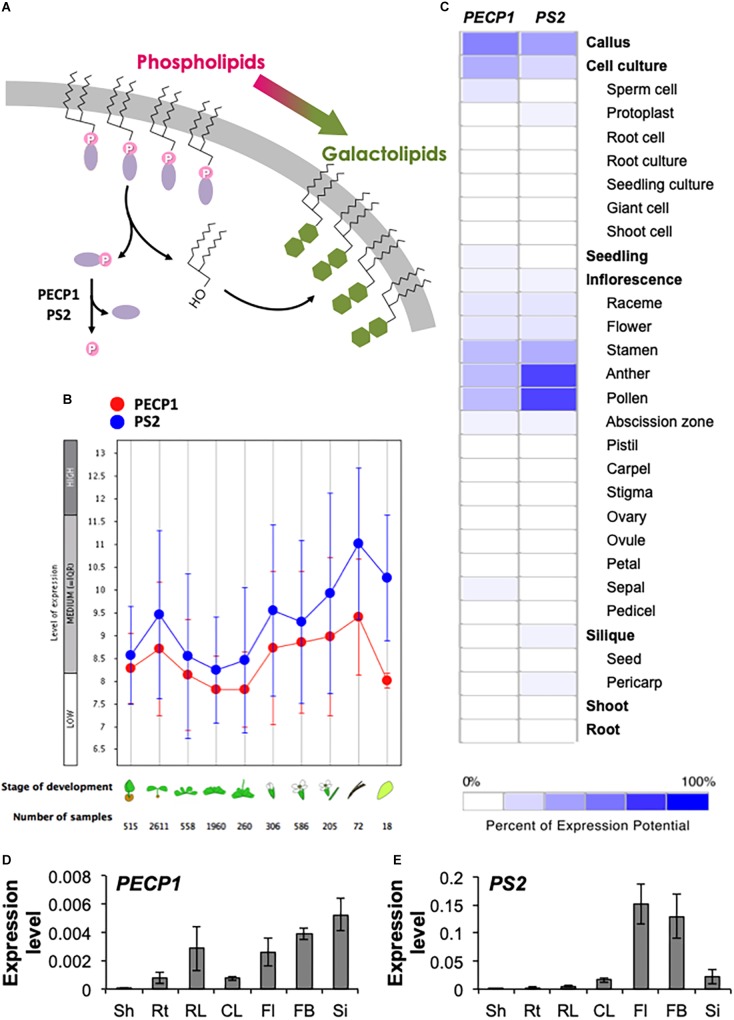
Gene expression pattern of *PECP1* and *PS2*. **(A)** Schematic representation of the membrane lipid remodeling under phosphate starvation and the reaction catalyzed by PECP1 and PS2. Purple ellipse, polar head group; pink circle, phosphate group; green hexagon, galactose. **(B)** Developmental stage-specific expression patterns of *PECP1* (red) and *PS2* (blue). Stage of development from left to right; germinating seed, seedling, young rosette, developed rosette, bolting rosette, young flower, developed flower, flower and siliques, mature siliques, and senescence. “HIGH,” “MEDIUM,” and “LOW” expression was calculated by microarray assay collected by GENEVESTIGATOR. **(C)** Heat map of tissue-specific pattern of *PECP1* and *PS2* generated by GENEVESTIGATOR. **(D,E)** Expression levels of *PECP1* **(D)** and *PS2* **(E)** analyzed by qRT-PCR with cDNA prepared from the different tissues of wild type. Data are mean ± SD from three biological replicates and three technical replicates. Values were normalized to *ACT2*. Sh, shoot; Rt, root; RL, rosette leaf; CL, cauline leaf; Fl, mature flower; FB, flower bud; Si, silique.

Phosphorus is an essential nutrient for plant growth and development. Due to limited availability in many soils, metabolic change toward utilization of plant internal phosphate reserve is an important response to circumvent phosphate starvation. Phospholipids have been focused as a major reserve of internal phosphate due to its abundance; however, they play critical roles in the construction and maintenance of cellular membranes. Indeed, a number of evidence indicates that phospholipid biosynthesis is essential for plant function (Nakamura, 2017). During phosphate starvation, a metabolic conversion termed the membrane lipid remodeling (Nakamura, 2013) occurs that replaces phosphate-containing polar head group of phospholipids with non-phosphorus galactose group, thereby converting the membrane lipid content from phospholipids to galactolipids presumably to cope with phosphate starvation (Figure 1A). Although gene knockout study that impedes the conversion process suggests that this metabolic remodeling is important in plant phosphate starvation tolerance (Nakamura et al., 2009), there is lack of evidence as to whether polar head group-derived phosphate is indeed utilized as a phosphate source. This is because the identity and function of phosphatase(s) that dephosphorylates the polar head group has remained enigmatic in plants.

*Phosphate starvation-induced gene 2* (*PS2*) is a commonly used marker for phosphate starvation response in *Arabidopsis* (Chandrika et al., 2013). A biochemical study showed that PS2 encodes a functional HAD-like phosphatase *in vitro*, for which the authors designated PPsPase1 (May et al., 2011). Later, a close homolog of PS2/PPsPase1, named PECP1, was found to be a phosphatase that dephosphorylates major polar head group of phospholipids such as phosphoethanolamine (PEtn) and phosphocholine (PCho) *in vitro* (May et al., 2012). Although PECP1 but not PS2/PPsPase1 hydrolyzes the phospholipid polar head group *in vitro*, gene knockout study of the *pecp1-1 ps2-3* double mutant (Angkawijaya and Nakamura, 2017) as well as *in planta* overexpression study (Hanchi et al., 2018) suggest that both are redundant in dephosphorylating the polar head group *in vivo*. Despite a possible overlapping function of two closely related phosphatases, comparative study on their tissue expression profiles and subcellular localization has not been reported. Besides, *in vivo* overexpression study of these enzymes toward PEtn dephosphorylation needs validation to clarify whether designated reaction product is indeed accumulated *in planta* following the overexpression. Thus, possibly differential function of these phosphatases remains obscure *in vivo*.

In this report, we investigated spatiotemporal expression profiles of PECP1 and PS2 proteins by constructing transgenic plants harboring functional translational promoter–reporter fusion system. Using GUS reporter, we showed the expression patterns of PECP1 and PS2 in different tissues and in response to phosphate starvation. The use of Venus fluorescent reporter revealed that both are localized at the ER. *In vivo* overexpression study indicates that their activity toward PEtn may be different *in vivo*. We suggest that PECP1 and PS2 are ER-localized phosphatases that show similar expression pattern yet have a distinct substrate specificity *in vivo.*

## Materials and Methods

### Plant Materials and Growth Conditions

*Arabidopsis thaliana* (ecotype; Columbia-0) was used throughout the study. Plants were grown under long-day (16 h light/8 h dark) photoperiodic condition at 22°C with light intensity of 75 μmol m^−2^ s^−1^. For plate culture, Murashige and Skoog (MS) medium was used at half-strength concentration (Murashige and Skoog, 1962). The *pecp1-1 ps2-3* plant was as described previously (Angkawijaya and Nakamura, 2017). For phosphate starvation experiments, medium was prepared according to previous publication (Angkawijaya et al., 2017).

### Vector Construction and Plant Transformation

Genomic sequence of *PECP1* (2,636 bp) was amplified by PCR from *A. thaliana* wild-type genomic DNA using the primers YN1397/YN1398 (see Supplemental Table 1 for the sequences of primers used hereafter). The PCR products were cloned into pENTR/D-TOPO plasmid (Invitrogen, Thermo Fisher Scientific, Waltham, MA) to obtain pYL21 (*pENTR_ProPECP1:PECP1*). To create either *ProPECP1:PECP1-GUS* or *ProPECP1:PECP1-Ven*, the *Sfo*I site was introduced 5′ to the stop codon of pYL21 by PCR-based site-directed mutagenesis (Sawano and Miyawaki, 2000) with a primer YN1404 to obtain pYL23. The GUS or Venus cassettes were inserted into the *Sfo*I site of this plasmid to generate pFG7 and pFG8, respectively.

To create *Pro35S:PECP1*, 840 bp of the open reading frame (ORF) for *PECP1* was amplified by PCR with the primers FG21 and FG22. To create this construct, *Sal*I site was inserted before the *Mlu*I site of pYN2047 (Lin et al., 2015) by PCR-based site-directed mutagenesis (Sawano and Miyawaki, 2000) with the primer PP68 to obtain pAA044. The ORF fragment was then cloned into the *Sal*I and *Eco*RI sites of pAA044 to obtain pFG14.

The obtained entry vector plasmids pFG7, pFG8, and pFG14 were recombined into the pKGW destination vector using LR Clonase (Invitrogen, Thermo Fisher Scientific, Waltham, MA) to generate pFG10, pFG11, and pFG5, respectively (Karimi et al., 2002). The generated plasmid was transduced into the *pecp1-1 ps2-3* by *Agrobacterium tumefaciens*-mediated transformation. Twenty-four T_1_ plants that showed resistance when grown on 1/2 MS plate containing Kanamycin were selected. To distinguish transgenic *PECP1* from endogenous *PECP1*, the following primers were designed and used; *ProPECP1:PECP1-GUS* (FG15/KK98), *ProPECP1:PECP1-Ven* (FG15/KK104), and *Pro35S:PECP1* (KK97/FG23). Transgenic plant lines used for the observation at T_2_ generation were: *ProPECP1:PECP1-GUS*, lines #10 and #17; *ProPECP1:PECP1-Ven*, lines #9 and #10; and *Pro35S:PECP1*, lines #2 and #5.

Genomic sequence of *PS2* (5,259 bp) was amplified by PCR from *A. thaliana* wild-type genomic DNA using the primers PK66/PK10. The PCR products were cloned into pENTR/D-TOPO plasmid to obtain pPK1 (*pENTR_ProPS2:PS2*). To create either *ProPS2:PS2-GUS* or *ProPS2:PS2-Ven*, the *Sfo*I site was introduced 5′ to the stop codon of pPK01 by PCR-based site-directed mutagenesis (Sawano and Miyawaki, 2000) with a primer PK11 to obtain pPK02. The GUS or Venus cassettes were inserted into the *Sfo*I site of this plasmid to generate pPK07 and pPK08, respectively.

To create *Pro35S:PS2*, 888 bp of *PS2* ORF was amplified by PCR with the primers PK52 and PK54. The ORF fragment was then cloned into the *Sal*I and *Xba*I sites of pAA044 to obtain pPK04.

The obtained entry vector plasmids pPK07, pPK08, and pPK04 were recombined into the pKGW destination vector using LR Clonase (Invitrogen, Thermo Fisher Scientific, Waltham, MA) to generate pPK09, pPK15, and pPK10, respectively (Karimi et al., 2002). The generated plasmids were transduced into the *pecp1-1 ps2-3* by *A. tumefaciens*-mediated transformation. Twenty-four T_1_ plants that showed resistance when grown on 1/2 MS plate containing Kanamycin were selected. To distinguish transgenic *PS2* from endogenous *PS2*, the following primers were designed and used; *ProPS2:PS2-GUS* (PK13/KK98), *ProPS2:PS2-Ven* (PK13/KK104), and *Pro35S:PS2* (PK13/CH72). Transgenic plant lines used for the observation at T_2_ generation were: *ProPS2:PS2-GUS*, lines #8 and #22; *ProPS2:PS2-Ven*, lines #1 and #7; and *Pro35S:PS2*, lines #1, #3 and #6.

### RNA Extraction and Quantitative RT-PCR

Total RNA was isolated from different tissues (shoot and root, 7-day-old seedlings grown on MS medium; rosette leaf, 3-week-old soil-grown plants; the rest of tissues, 5-week-old soil grown plants) as previously described (Lin et al., 2015). Quantitative RT-PCR (qRT-PCR) was performed with the following primer; *PECP1* (FG24/FG25), *PS2* (FG28/FG29), and *ACTIN2* (KK129/KK130) as a control. Data are mean ± SD from three biological replicates, with three technical replicates.

### Microscopy Analysis

GUS expression assay of PECP1-GUS and PS2-GUS proteins were performed at various growth stages and at different phosphate availability with histochemical GUS staining as described previously (Lin et al., 2015).

Venus fluorescence observation of Pi-starved *ProPECP1:PECP1-Ven pecp1-1 ps2-3* and *ProPS2:PS2-Ven pecp1-1 ps2-3* seedlings was performed under a confocal microscope (LSM 510 Meta; Carl Zeiss, Jena, Germany) equipped with Plan-Apochromat 20×/0.8-NA, and Plan-Apochromat 10×/0.45-NA. For staining of the plasma membrane or ER, seedlings were immersed in 5 μg/ml of FM 4-64 (F34653, Thermo Fisher Scientific, Waltham, MA) for 2 min or immersed in 2 μM of the ER-Tracker Red dye (E34250, Thermo Fisher Scientific, Waltham, MA) for 30 min, prior to confocal microscopic observation. Images were captured by use of LSM 510 v3.2 (Carl Zeiss, Jena, Germany) with filters for Venus (514 nm laser, 520–555 nm band-pass), for FM 4-64 (543 nm laser, 560–615 nm band-pass), and for ER-Tracker Red dye (543 nm laser, 560 nm long pass).

### Metabolite Analysis

Extraction of ethanolamine (Etn) was conducted according to previous publications with 10 μL of 10 μM L-Norvaline (Sigma-Aldrich, St. Louis, MO) as an internal standard (Tannert et al., 2018). Analysis of derivatized Etn was performed with Agilent 1260 HPLC-DAD system (Agilent Technologies, Santa Clara, CA) equipped with a binary pump system (G1312B), degasser (G1322A), robotic autosampler (G1329B), column thermostat (G1316A), diode array HPLC detector (G1315D), and Poroshell 120 HPH-C18 column (size 4.6 mm × 100 mm, 2.7 μm). The flowrate was 0.62 μl/min using a gradient of mobile A (10 mM Na_2_HPO_4_, 10 mM Na_2_B_4_O_7_, and 5 mM NaN_3_; pH 8.2) and mobile B (45% acetonitrile/45% methanol/10% water). Gradient run time was set to 12 min with the following profile: 0–0.2 min isocratic 2% B, 0.2–10 min 2–57% B, 10–10.1 min 57–100% B, 10.1–11.7 min isocratic 100% B, 11.7–11.8 min 100–2% B, 11.8–12 min 2% B. The derivatization of Etn was conducted according to (Bartolomeo and Maisano, 2006). Polar glycerolipid analysis was conducted as described (Angkawijaya et al., 2017).

## Results

### Developmental- and Tissue-Specific Expression Patterns of PECP1 and PS2

To investigate the developmental- and tissue-specific expression patterns of *PECP1* and *PS2*, we first analyzed public transcript database with GENEVESTIGATOR. As shown in Figure 1B, *PECP1* and *PS2* showed similar developmental stage-specific expression profiles; the highest expression level was found in mature siliques, and seedlings and flowers showed relatively higher expression levels as compared to the other stages of tissues examined. Further analysis of the tissue-specific expression pattern showed that both are highly expressed in the male reproductive organ including stamen, anther, and pollen (Figure 1C).

Next, to confirm this profile, we extracted total RNA from seven different tissues in *A. thaliana* wild-type plants and analyzed the relative expression levels of *PECP1* and *PS2* (Figure 1D,E). Despite that these two genes showed similar levels of expression in different developmental stages (Figure 1B), our qRT-PCR data showed that *PECP1* had much lower expression level than *PS2* in floral organs (Figure 1D,E), which is in agreement with the data in Figure 1C. However, in other tissues examined, *PS2* showed higher expression levels than *PECP1*; in shoot (2.0 × 10^−4^ vs. 4.0 × 10^−5^), root (2.0 × 10^−3^ vs. 7.6 × 10^−4^), rosette leaf (5.1 × 10^−3^ vs. 2.9 × 10^−3^), cauline leaf (1.6 × 10^−2^ vs. 7.5 × 10^−4^), mature flower (1.5 × 10^−1^ vs. 2.6 × 10^−3^), flower bud (1.3 × 10^−1^ vs. 3.9 × 10^−3^), and silique (2.2 × 10^−2^ vs. 5.3 × 10^−3^). These tissue expression patterns largely agreed with the result of transcript database analysis (Figure 1B,C).

### Tissue-Specific Expression Pattern of PECP1 and PS2

**FIGURE 2:**
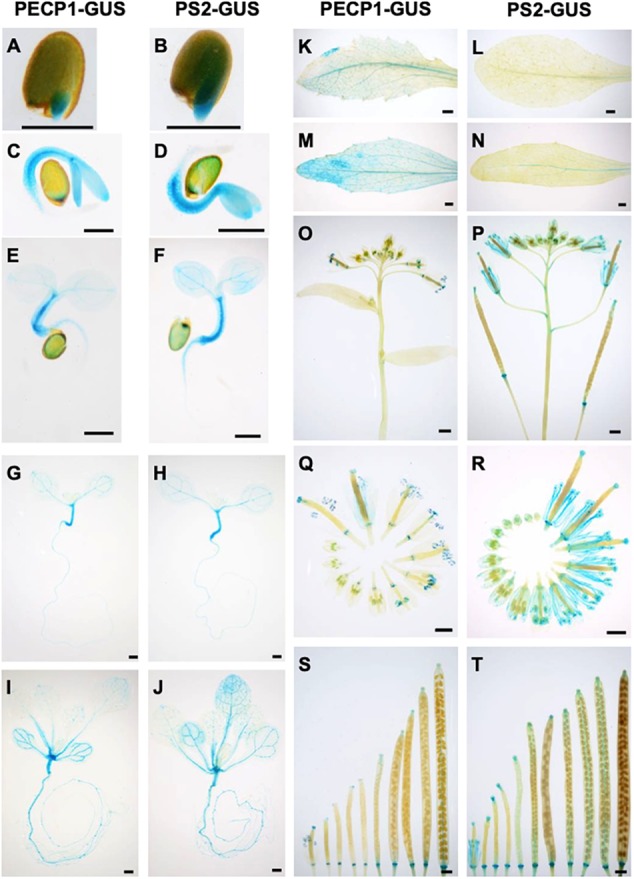
Tissue-specific expression of PECP1-GUS and PS2-GUS in *A. thaliana ProPECP1:PECP1-GUS pecp1-1 ps2-3* and *ProPS2:PS2-GUS pecp1-1 ps2-3* plants. **(A,C,E,G,I,K,M,O,Q,S)** GUS staining of *ProPECP1:PECP1-GUS pecp1-1- ps2-3* line #10. **(B,D,F,H,J,L,N,P,R,T)** GUS staining of *ProPS2:PS2-GUS pecp1-1 ps2-3* line #8. **(A,B)** 1 day, **(C,D)** 2 days, **(E,F)** 3 days, **(G,H)** 7 days, and **(I,J)** 14-days-old seedlings. **(K,L)** Rosette leaf. **(M,N)** Cauline leaf. **(O,P)** Inflorescence. **(Q,R)** Flowers at different developmental stages. **(S,T)** Developing siliques. Bars = 0.5 mm in panels **(A)–(H)** and 1 mm in panels **(I)–(T)**.

To study the protein expression profiles of PECP1 and PS2, we constructed transgenic plants that express PECP1-GUS or PS2-GUS fusion protein by their own promoters in the *pecp1-1 ps2-3* double mutant background. As shown in Figure 2, both were highly expressed in hypocotyls in 1–7 days old germinating seedlings (Figure 2A–H). At 14 days old, GUS staining was observed in leaf vasculatures and roots (Figure 2I,J). The GUS staining pattern was highly similar between PECP1 and PS2 by 14 days old. However, some difference in staining pattern was observed at later stages. In rosette and cauline leaves, no PS2-GUS expression was observed while PECP1-GUS staining was found in leaf veins (Figure 2K–N). In inflorescences, a faint GUS staining was seen in the node for PS2-GUS but not PECP1-GUS (Figure 2O,P). During flower development, both showed staining at early stages of anther development. At later stages, PS2-GUS staining was observed mainly in anther filaments while PECP1-GUS staining remained specifically in anthers (Figure 2Q,R). Neither one showed staining in developing siliques (Figure 2S,T). These staining patterns were confirmed with another independent transgenic line for each GUS reporter (Supplementary Figure 1), so PECP1 and PS2 have overlapping tissue specificity in seedlings but distinct profile at later developmental stages.

### Expression Pattern of PECP1 and PS2 in Response to Phosphate Starvation

**FIGURE 3:**
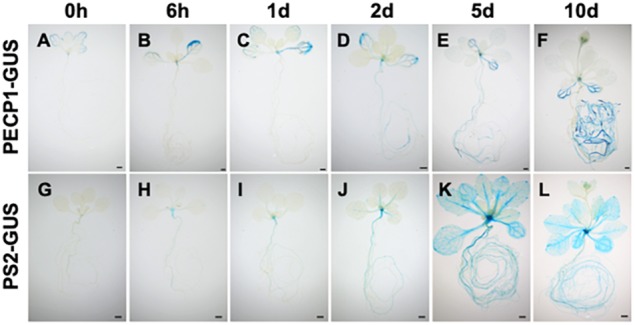
Time-course profiles of the expression patterns of PECP1-GUS and PS2-GUS upon phosphate starvation. Seedlings of *ProPECP1:PECP1-GUS pecp1-1 ps2-3* line #10 **(A–F)** and *ProPS2:PS2-GUS pecp1-1 ps2-3* line #8 **(G–L)** were stained 0 day **(A,G)**, 6 h **(B,H)**, 1 day **(C,I)**, 2 days **(D,J)**, 5 days **(E,K)**, 10 days **(F,L)** after transfer to phosphate-starved media. Bars = 1 mm.

To investigate how expression of PECP1 and PS2 is induced by phosphate starvation, we performed time-course GUS staining at different time points of phosphate starvation after transferring 10-day-old phosphate-replete seedlings of *ProPECP1:PECP1-GUS pecp1-1 ps2-3* and *ProPS2:PS2-GUS pecp1-1 ps2-3* to phosphate-starved condition. As shown in Figure 3, no obvious change in the GUS staining pattern was observed during the first 24 h following phosphate starvation (Figure 3A–C,G–I). At 2 days after phosphate starvation, however, a slightly enhanced GUS staining was observed in the root and leaf vasculature (Figure 3D,J). At 5 days, a markedly enhanced GUS staining was observed in PS2-GUS but not PECP1-GUS (Figure 3E,K), which showed further enhanced staining by 10 days after phosphate starvation (Figure 3F,L). This expression pattern was confirmed with another independent line for each GUS reporter (Supplementary Figure 2). Thus, our histochemical observation showed temporal induction pattern of PECP1 and PS2 proteins in response to phosphate starvation.

### PECP1 and PS2 Were Localized at the ER

**FIGURE 4:**
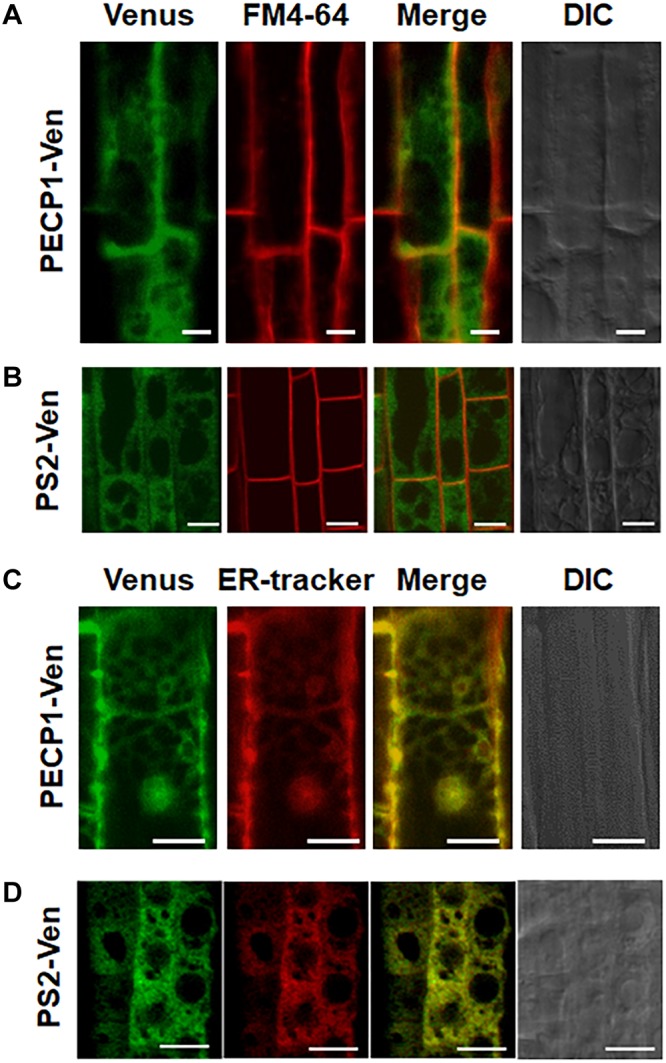
Subcellular localization of PECP1-Ven and PS2-Ven in phosphate-starved root cells by confocal microscope observation. Seedling roots of phosphate-starved *ProPECP1:PECP1-Ven pecp1-1 ps2-3* line #9 **(A,C)** and *ProPS2:PS2-Ven pecp1-1 ps2-3* line #1 **(B,D)** were observed for the overlap of Venus florescence signal with staining of a plasma membrane marker FM4-64 **(A,B)** or an ER marker ER-Tracker **(C,D)**. Expression of PECP1-Ven **(A,C)** and PS2-Ven **(B,D)** with staining pattern of FM4-64 for plasma membrane marker **(A,B)** and ER-tracker **(C,D)** were merged. Bars = 10 μm.

To investigate subcellular localization of PECP1 and PS2, we constructed transgenic plants harboring *ProPECP1:PECP1-Ven* or *ProPS2:PS2-Ven* in the *pecp1-1 ps2-3* background. We observed Ven fluorescent signal in the root cells. Fluorescent signal of neither PECP1-Ven (Figure 4A) nor PS2-Ven (Figure 4B) overlapped with the staining of plasma membrane marker dye (FM4-64). However, a clear overlap was observed with the ER marker dye (ER Tracker) for both PECP1-Ven (Figure 4C) and PS2-Ven (Figure 4D). This expression pattern was confirmed with another independent line for each Ven reporter (Supplementary Figure 3). These results suggest that both PECP1 and PS2 are localized at the ER.

### Involvement of PECP1 and PS2 in Ethanolamine Production *in vivo*

**FIGURE 5:**
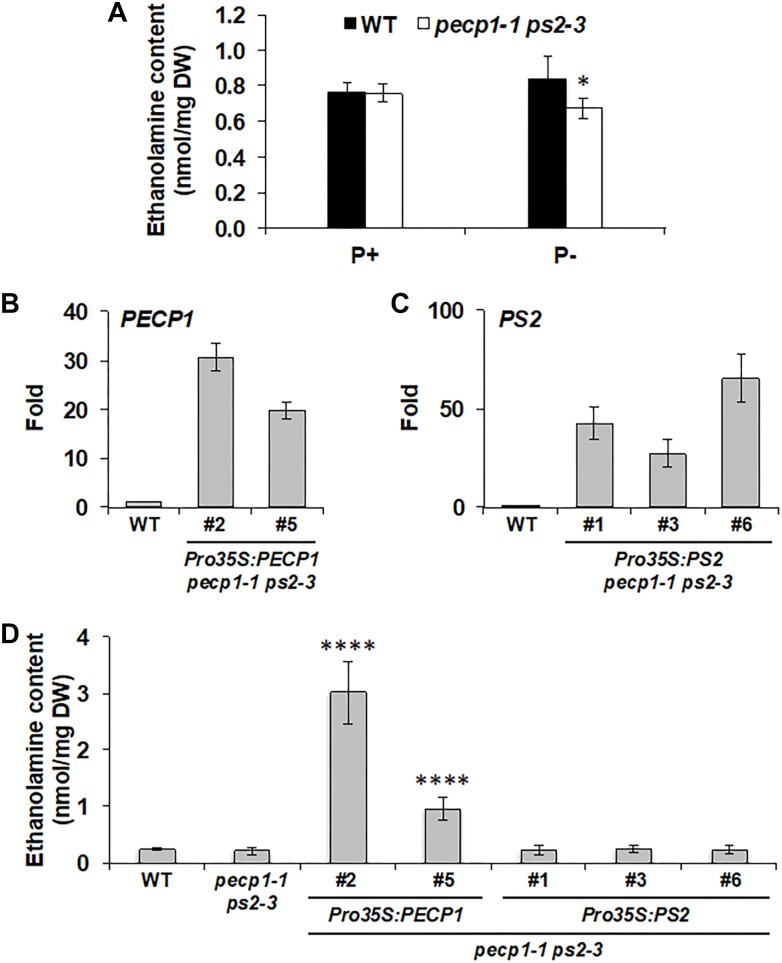
Effect of PECP1 and PS2 on ethanolamine (Etn) content *in vivo*. **(A)** Etn content in 20-day-old seedlings of the wild type (WT; black bars) and *pecp1-1 ps2-3* (white bars) raised for the first 10 days in phosphate-replete media then transferred to phosphate-replete (P+) or –starved (P–) media for another 10 days of culture. **(B,C)** Relative expression of *PECP1* **(B)** and *PS2* **(C)** among representative transgenic plant lines harboring either *Pro35S:PECP1* or *Pro35S:PS2* as compared with WT. **(D)** Amount of Etn in 20 days old rosette leaves of wild type, *pecp1-1 ps2-3*, *Pro35S:PECP1 pecp1-1 ps2-3* lines #2 and #5, and *Pro35:PS2 pecp1-1 ps2-3* lines #1, #3, and #6. Data are mean ± SD from at least six biological replicates. The asterisks indicate significance by Student’s *t*-test (^∗∗∗∗^*p* < 0.0001; ^∗^*p* < 0.05).

During phosphate starvation, PC is hydrolyzed by phosphate starvation-inducible non-specific phospholipase C4 and 5 (NPC4 and NPC5; Nakamura et al., 2005; Gaude et al., 2008), that provide DAG and PCho. While DAG is the substrate for DGDG biosynthesis, PCho may be dephosphorylated by phosphate starvation-inducible PECP1 and PS2 to produce phosphate and choline (Cho) (Angkawijaya and Nakamura, 2017). Indeed, the double mutant *pecp1-1 ps2-3* showed reduced Cho content under phosphate starvation (Angkawijaya and Nakamura, 2017). Moreover, overexpression of PECP1 (Hanchi et al., 2018; Tannert et al., 2018) or PS2 (Hanchi et al., 2018) decreased PCho content *in planta*. However, phosphatidylethanolamine (PE) content also decreases in response to phosphate starvation (Essigmann et al., 1998) and NPC4 and 5 can hydrolyze PE *in vitro* (Nakamura et al., 2005; Gaude et al., 2008). In this context, PEtn may be another available substrate for PECP1 and PS2 *in vivo*. According to *in vitro* study, PECP1 dephosphorylates PEtn (May et al., 2012). *In vivo*, overexpression of *PECP1* or *PS2* decreases PEtn content (Hanchi et al., 2018). However, it is unknown whether changes in PEtn contents are due to dephosphorylation activity because Etn content was not analyzed. To examine whether PECP1 and PS2 are involved in dephosphorylating PEtn *in vivo*, we first analyzed Etn content in the seedlings of *pecp1-1 ps2-3*. Under phosphate-replete condition, no change was observed in Etn content between wild type and the mutant (Figure 5A). Under phosphate-starved condition, however, a slight but significant reduction was detected (Figure 5A), which indicates that PECP1 and PS2 may be involved in the dephosphorylation of PEtn *in vivo* under phosphate starvation. Next, to test whether the GUS and Ven fusion constructs we used for the expression study are functional *in vivo*, we quantified Etn contents in the seedlings of four transgenic lines characterized in Figure 2–4; *ProPECP1:PECP1-Ven pecp1-1 ps2-3* line #9, *ProPS2:PS2-Ven pecp1-1 ps2-3* line #1, *ProPECP1:PECP1-GUS pecp1-1 ps2-3* line #10, and *ProPS2:PS2-GUS pecp1-1 ps2-3* line #8. As shown in Supplementary Figure 4, the lower Etn content in the *pecp1-1 ps2-3* was rescued in all the transgenic lines. Moreover, we found that all the second transgenic lines used for the confirmation in Supplementary Figures S1–S3, except *ProPS2:PS2-GUS pecp1-1 ps2-3* line #22, also rescued the lower Etn content in the *pecp1-1 ps2-3.* These results indicate that these transgenes are functional *in vivo*.

To further confirm the enzymatic function, we constructed transgenes that express PECP1 or PS2 by cauliflower mosaic virus 35S promoter (Pro35S) and transduced them into the *pecp1-1 ps2-3*. We obtained 24 lines each for *Pro35S:PECP1 pecp1-1 ps2-3* and *Pro35S:PS2 pecp1-1 ps2-3*, and screened 2–3 transgenic lines each with the highest transcript levels of encoded genes (Figure 5B,C). Using these lines, we compared the Etn contents under phosphate-replete growth condition. As shown in Figure 5D, two independent lines of *Pro35S:PECP1 pecp1-1 ps2-3* both increased Etn contents significantly. However, no significant increase was observed among three independent transgenic lines of *Pro35S:PS2 pecp1-1 ps2-3* compared with wild type. These results suggest that PECP1 has major contribution to produce Etn *in vivo*.

Finally, we tested a possible impact of *PECP1* and *PS2* loss-of-function and *PECP1* overexpression on PC and PE contents. We performed membrane glycerolipid analysis in the seedlings of wild type, *pecp1-1 ps2-3*, and *Pro35S:PECP1 pecp1-1 ps2-3* (lines #2 and #5). As shown in Supplementary Figure 5, no significant change was observed for the glycerolipid contents (Supplementary Figures 5A,B). We also analyzed the fatty acid composition in each glycerolipid class analyzed; however, only marginal change was observed in 18:3 content of PC and 16:1 content of phosphatidylglycerol (PG) (Supplementary Figure 5C). Thus, PECP1 and PS2 have little impact on the membrane glycerolipid contents.

## Discussion

Membrane lipid remodeling is an important metabolic response for plants to cope with phosphate starvation (Nakamura, 2013). In phospholipid hydrolysis by NPC, the metabolic fate of phosphate-containing head group has been enigmatic while that of DAG is for the precursor of DGDG biosynthesis. A series of recent reports about *in vitro* (May et al., 2011, 2012) and *in vivo* (Angkawijaya and Nakamura, 2017; Hanchi et al., 2018; Tannert et al., 2018) characterization on PECP1 and PS2 suggest their possible involvement in the dephosphorylation of the head group of phospholipids, a crucial reaction to release scarce phosphate originated from the membrane phospholipids (Figure 1A). Here, our reports provide expression profiles of these phosphatases at tissue and subcellular levels. Moreover, metabolite analysis of transgenic plants overexpressing *PECP1* or *PS2* revealed substrate preference of these phosphatases *in vivo*.

Tissue-specific expression study with GUS reporter system demonstrated that both PECP1 and PS2 are expressed in rather limited type of tissues under normal condition (Figure 2). However, upon phosphate starvation, the expression is markedly induced in almost the entire seedling (Figure 3), which indicates that PECP1 and PS2 are phosphate starvation-inducible proteins in different seedling tissues. Indeed, levels of Cho and Etn in the *pecp1-1 ps2-3* were affected only under phosphate starvation (Figure 5A; Angkawijaya and Nakamura, 2017). Thus, these phosphatases may have redundant function *in vivo* under phosphate starvation (Angkawijaya and Nakamura, 2017; Hanchi et al., 2018).

What is the substrate specificity of PECP1 and PS2 *in vivo*? Enzymatic characterization *in vitro* shows that PECP1 prefers PEtn than PCho for its substrate (May et al., 2012). *In vivo*, overexpression of PECP1 in the *pecp1-1 ps2-3* increased Etn content (Figure 5D). Although *pecp1-1* showed no reduction in Etn level, reduced PEtn phosphatase activity was observed in *pecp1-1* (Tannert et al., 2018). No reduction in PCho phosphatase activity was observed in *pecp1-1* plants (Tannert et al., 2018). Thus, PECP1 prefers PEtn than PCho both *in vitro* and *in vivo*. Regarding PS2, *in vitro* enzyme activity assay with recombinant PS2 protein shows that neither PEtn nor PCho is a substrate under their assay condition (May et al., 2011). Our result of Etn measurement in phosphate-replete *Pro35S:PS2 pecp1-1 ps2-3* plants showed no changes (Figure 5D). Nevertheless, phosphate-starved double mutants of *PECP1* and *PS2* showed an increase in PEtn and PCho (Hanchi et al., 2018) as well as decreases in Etn (Figure 5A) and Cho (Angkawijaya and Nakamura, 2017), which suggest functional redundancy between PECP1 and PS2. Although no clear evidence is available to address this discrepancy, a possible scenario is that PS2 might require some modification to dephosphorylate PEtn or PCho under phosphate starvation.

ER localization of PECP1 and PS2 (Figure 4) may facilitate access to the substrate, since ER is the major site of phospholipid metabolism. Indeed, NPC5 is ER-localized and is involved in membrane lipid remodeling under phosphate starvation (Gaude et al., 2008). It is possible that a reaction product of NPC activity may be further dephosphorylated by PECP1 and PS2 to liberate free phosphate. Besides, additional pathway(s) may produce the substrate for these phosphatases. For example, phospho-base *N*-methyltransferase1 and 2 (PMT1 and 2) are transcriptionally induced by phosphate starvation (Misson et al., 2005). On the other hand, levels of the reaction products Etn and Cho may be influenced by contribution of some other pathways related to Etn and Cho production. Some of phospholipase D and glycerophosphodiester phosphodiesterase, which produce Etn or Cho, are induced by phosphate starvation (Li et al., 2006; Gaude et al., 2008; Cheng et al., 2011). This postulates an increase in Etn and Cho contents. It is possible that the decreased production of Etn and Cho in the *pecp1-1 ps2-3* may be compensated by these pathways. Future study is anticipated to elucidate the complex interplay of metabolic pathways to acquire free phosphate from membrane phospholipids under phosphate starvation.

## Data Availability

All datasets generated for this study are included in the manuscript and/or the Supplementary Files.

## Author Contributions

AA, AN, VN, and FG performed the experiments, analyzed the data, and wrote the article. YN conceived the research, supervised AA, AN, VN, and FG’s experiments, and wrote the article. All authors commented on the article and approved the contents.

## Conflict of Interest Statement

The authors declare that the research was conducted in the absence of any commercial or financial relationships that could be construed as a potential conflict of interest.

## Abbreviations

Cho: choline
DAG: diacylglycerol
Etn: ethanolamine
PC: phosphatidylcholine
PCho: phosphocholine
PE: phosphatidylethanolamine
PEtn: phosphoethanolamine
PECP1: phosphoethanolamine/phosphocholine phosphatase 1
PS2: phosphate starvation-induced gene 2.

## References

[B1] Angkawijaya A. E. Nakamura Y. (2017). Arabidopsis PECP1 and PS2 are phosphate starvation-inducible phosphocholine phosphatases. *Biochem. Biophys. Res. Commun.* 494 397–401. 10.1016/j.bbrc.2017.09.094 28942147

[B2] Angkawijaya A. E. Nguyen V. C. Nakamura Y. (2017). Enhanced root growth in phosphate-starved Arabidopsis by stimulating *de novo* phospholipid biosynthesis through the overexpression of LYSOPHOSPHATIDIC ACID ACYLTRANSFERASE 2 (LPAT2). *Plant Cell Environ.* 40 1807–1818. 10.1111/pce.12988 28548242

[B3] Bartolomeo M. P. Maisano F. (2006). Validation of a reversed-phase HPLC method for quantitative amino acid analysis. *J. Biomol. Tech.* 17 131–137. 16741240 PMC2291777

[B4] Chandrika N. N. Sundaravelpandian K. Yu S. M. Schmidt W. (2013). ALFIN-LIKE 6 is involved in root hair elongation during phosphate deficiency in Arabidopsis. *New Phytol.* 198 709–720. 10.1111/nph.12194 23432399

[B5] Cheng Y. Zhou W. El Sheery N. I. Peters C. Li M. Wang X. (2011). Characterization of the arabidopsis glycerophosphodiester phosphodiesterase (GDPD) family reveals a role of the plastid-localized AtGDPD1 in maintaining cellular phosphate homeostasis under phosphate starvation. *Plant J.* 66 781–795. 10.1111/j.1365-313X.2011.04538.x 21323773

[B6] Essigmann B. Guler S. Narang R. A. Linke D. Benning C. (1998). Phosphate availability affects the thylakoid lipid composition and the expression of *SQD1*, a gene required for sulfolipid biosynthesis in *Arabidopsis thaliana*. *Proc. Natl. Acad. Sci. U.S.A.* 95 1950–1955. 10.1073/pnas.95.4.1950 9465123 PMC19220

[B7] Gaude N. Nakamura Y. Scheible W.-R. Ohta H. Dörmann P. (2008). Phospholipase C5 (NPC5) is involved in galactolipid accumulation during phosphate limitation in leaves of Arabidopsis. *Plant J.* 56 28–39. 10.1111/j.1365-313X.2008.03582.x 18564386

[B8] Hanchi M. Thibaud M. C. Legeret B. Kuwata K. Pochon N. Beisson F. (2018). The phosphate fast-responsive genes *PECP1* and *PPsPase1* affect phosphocholine and phosphoethanolamine content. *Plant Physiol.* 176 2943–2962. 10.1104/pp.17.01246 29475899 PMC5884592

[B9] Karimi M. Inzé D. Depicker A. (2002). GATEWAY vectors for *Agrobacterium*-mediated plant transformation. *Trends Plant Sci.* 7 193–195. 10.1016/s1360-1385(02)02251-3 11992820

[B10] Li M. Welti R. Wang X. (2006). Quantitative profiling of Arabidopsis polar glycerolipids in response to phosphorus starvation. Roles of phospholipases Dζ1 and Dζ2 in phosphatidylcholine hydrolysis and digalactosyldiacylglycerol accumulation in phosphorus-starved plants. *Plant Physiol.* 142 750–761. 10.1104/pp.106.085647 16891548 PMC1586058

[B11] Lin Y. C. Liu Y. C. Nakamura Y. (2015). The choline/ethanolamine kinase family in Arabidopsis: essential role of CEK4 in phospholipid biosynthesis and embryo development. *Plant Cell* 27 1497–1511. 10.1105/tpc.15.00207 25966764 PMC4456650

[B12] May A. Berger S. Hertel T. Köck M. (2011). The *Arabidopsis thaliana* phosphate starvation responsive gene *AtPPsPase1* encodes a novel type of inorganic pyrophosphatase. *Biochim. Biophys. Acta* 1810 178–185. 10.1016/j.bbagen.2010.11.006 21122813

[B13] May A. Spinka M. Köck M. (2012). *Arabidopsis thaliana* PECP1 — Enzymatic characterization and structural organization of the first plant phosphoethanolamine/phosphocholine phosphatase. *Biochim. Biophys. Acta* 1824 319–325. 10.1016/j.bbapap.2011.10.003 22024570

[B14] Misson J. Raghothama K. G. Jain A. Jouhet J. Block M. A. Bligny R. (2005). A genome-wide transcriptional analysis using *Arabidopsis thaliana* Affymetrix gene chips determined plant responses to phosphate deprivation. *Proc. Natl. Acad. Sci. U.S.A.* 102:11934. 10.1073/pnas.0505266102 16085708 PMC1188001

[B15] Murashige T. Skoog F. (1962). A revised medium for rapid growth and bio assays with tobacco tissue cultures. *Physiol. Plant.* 15 473–497. 10.1111/j.1399-3054.1962.tb08052.x

[B16] Nakamura Y. (2013). Phosphate starvation and membrane lipid remodeling in seed plants. *Prog. Lipid Res.* 52 43–50. 10.1016/j.plipres.2012.07.002 22954597

[B17] Nakamura Y. (2017). Plant phospholipid diversity: emerging functions in metabolism and protein-lipid interactions. *Trends Plant Sci.* 22 1027–1040. 10.1016/j.tplants.2017.09.002 28993119

[B18] Nakamura Y. Awai K. Masuda T. Yoshioka Y. Takamiya K. Ohta H. (2005). A novel phosphatidylcholine-hydrolyzing phospholipase C induced by phosphate starvation in Arabidopsis. *J. Biol. Chem.* 280 7469–7476. 10.1074/jbc.m408799200 15618226

[B19] Nakamura Y. Koizumi R. Shui G. Shimojima M. Wenk M. R. Ito T. (2009). *Arabidopsis* lipins mediate eukaryotic pathway of lipid metabolism and cope critically with phosphate starvation. *Proc. Natl. Acad. Sci. U.S.A.* 106 20978–20983. 10.1073/pnas.0907173106 19923426 PMC2791602

[B20] Sawano A. Miyawaki A. (2000). Directed evolution of green fluorescent protein by a new versatile PCR strategy for site-directed and semi-random mutagenesis. *Nucleic Acids Res.* 28:E78. 10931937 10.1093/nar/28.16.e78 PMC108465

[B21] Tannert M. May A. Ditfe D. Berger S. Balcke G. U. Tissier A. (2018). Pi starvation-dependent regulation of ethanolamine metabolism by phosphoethanolamine phosphatase PECP1 in Arabidopsis roots. *J. Exp. Bot.* 69 467–481. 10.1093/jxb/erx408 29294054 PMC5853852

