# Supplementary material for: Expression Profiles of 2 Phosphate Starvation-Inducible Phosphocholine/Phosphoethanolamine Phosphatases, PECP1 and PS2, in Arabidopsis
Source: Front Plant Sci. 2019 May 29;10:662. doi: 10.3389/fpls.2019.00662 (PMC6549246; doi:10.3389/fpls.2019.00662)
Supplement: Supplementary file 1 [file Data_Sheet_1.pdf]

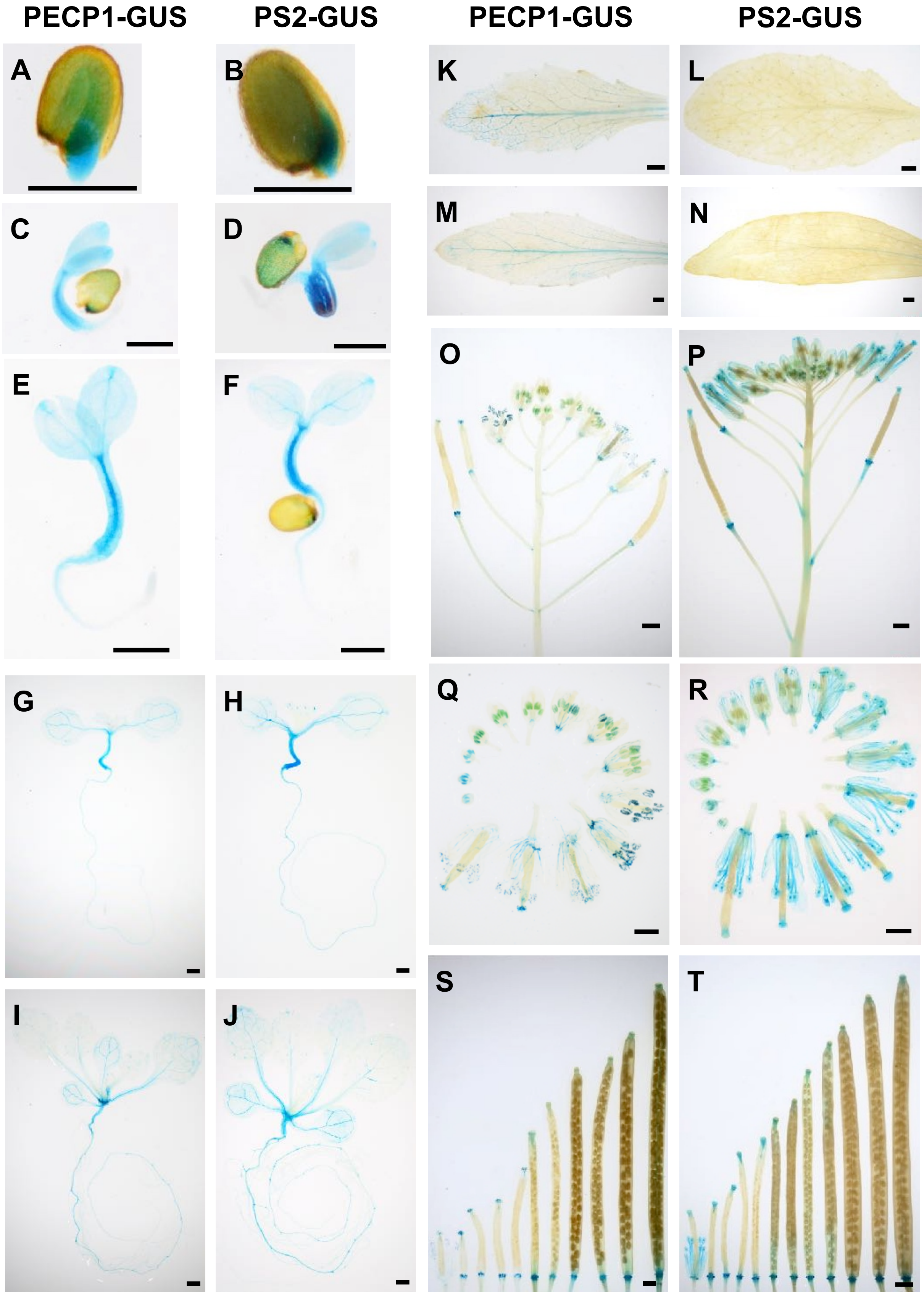

Supplementary Figure 1 | Tissue-specific expression of PECP1-GUS and PS2-GUS in *Arabidopsis thaliana ProPECP1:PECP1-GUS pecp1-1 ps2-3* and *ProPS2:PS2-GUS pecp1-1 ps2-3* plants. (A, C, E, G, I, K, M, O, Q, S) GUS staining of *ProPECP1:PECP1-GUS pecp1-1-ps2-3* line #17. (B, D, F, H, J, L, N, P, R, T) GUS staining of *ProPS2:PS2-GUS pecp1-1 ps2-3* line #22. (A, B) 1 d, (C, D) 2 d, (E, F) 3 d, (G, H) 7 d, and (I, J) 14-d-old seedlings. (K, L) Rosette leaf. (M, N) Cauline leaf. (O, P) Inflorescence. (Q, R) Flowers at different developmental stages. (S, T) Developing siliques. Bars= 0.5mm in (A) to (H) and 1mm in (I) to (T)

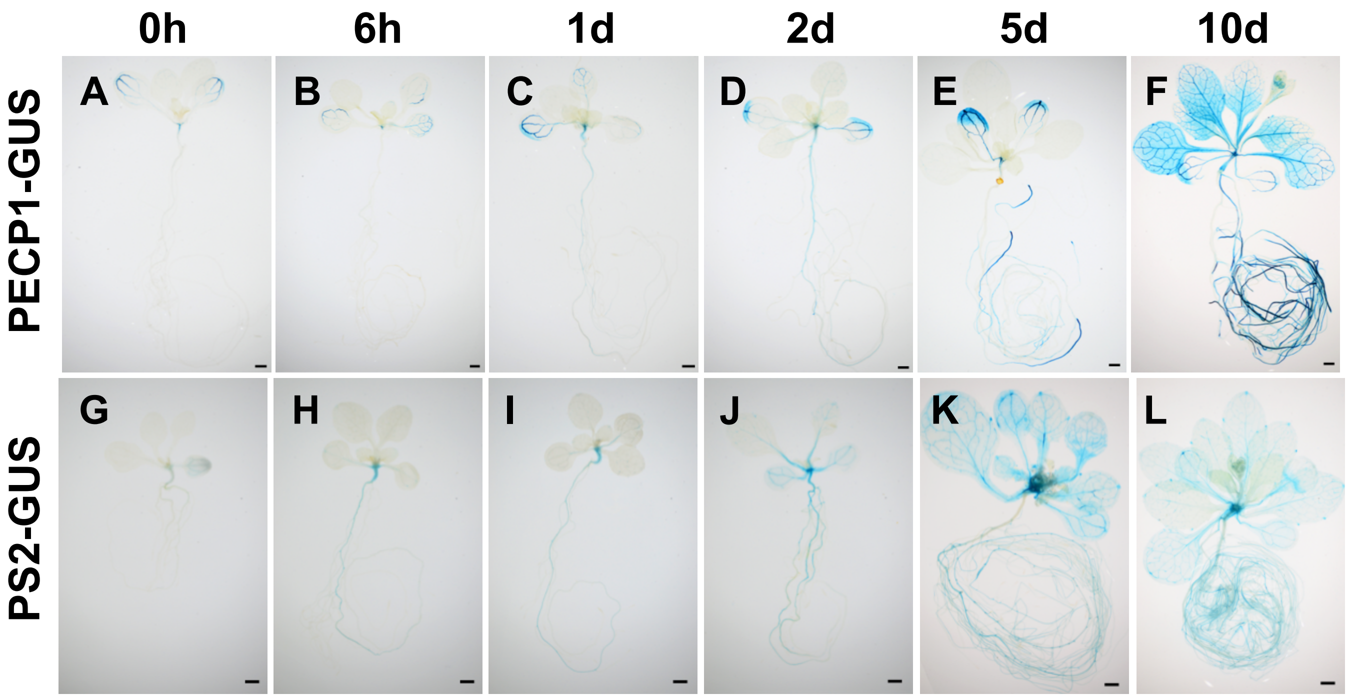

Supplementary Figure 2 | Time-course profiles of the expression patterns of PECP1-GUS and PS2-GUS upon phosphate starvation. Seedlings of *ProPECP1:PECP1-GUS pecp1-1 ps2-3* line #17 (A-F) and *ProPS2:PS2-GUS pecp1-1 ps2-3* line #22 (G-L) were stained 0 d (A and G), 6 h (B and H), 1 d (C and I), 2 d (D and J), 5 d (E and K), 10 d (F and L) after transfer to phosphate-starved media. Bars = 1 mm.

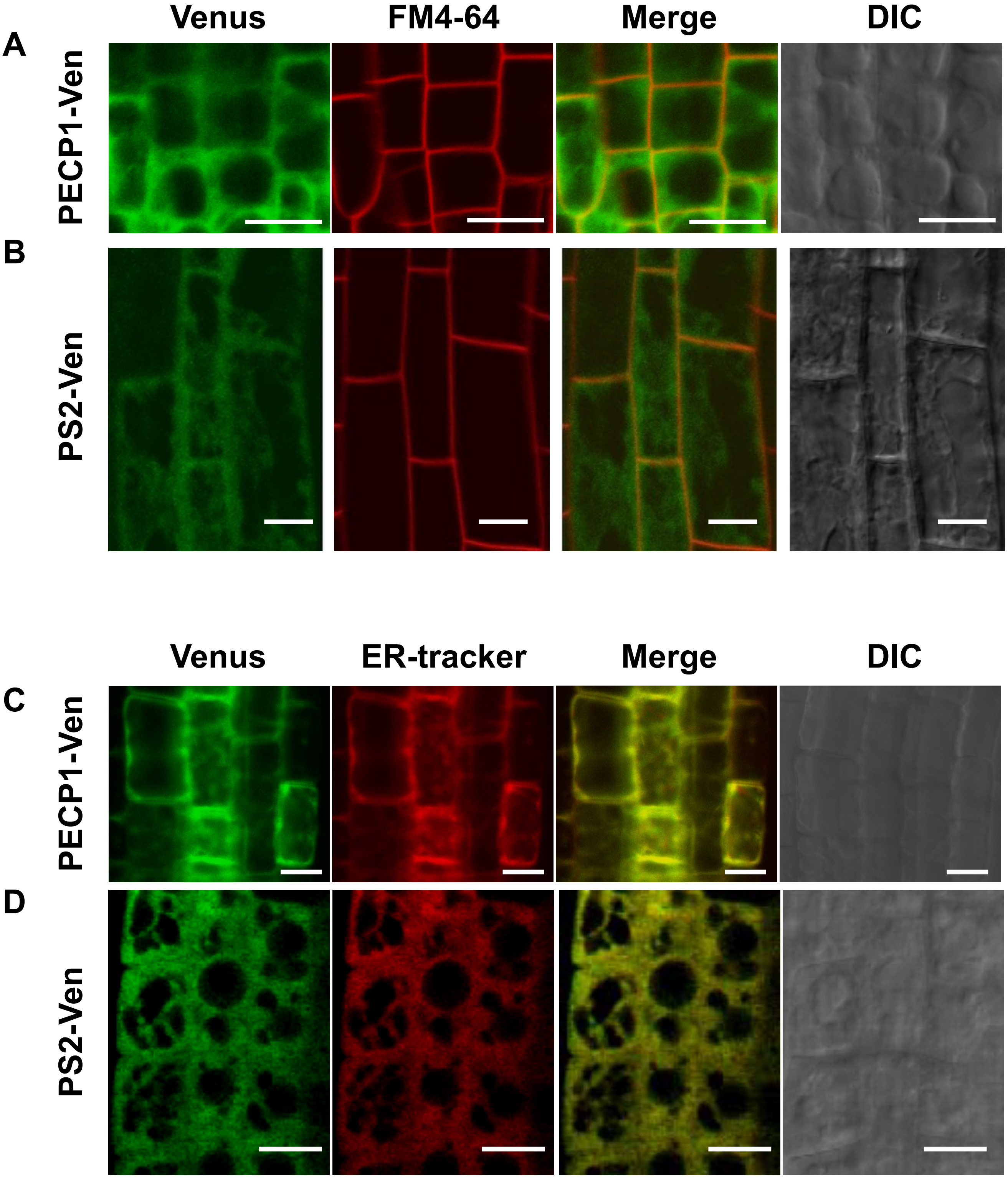

Supplementary Figure 3 | Subcellular localization of PECP1-Ven and PS2-Ven in phosphate-starved root cells by confocal microscope observation. Seedling roots of phosphate-starved *ProPECP1:PECP1-Ven pecp1-1 ps2-3* line #10 (A, C) and *ProPS2:PS2-Ven pecp1-1 ps2-3* line #7 (B, D) were observed for the overlap of venus florescence signal with staining of a plasma membrane marker FM4-64 (A, B) or an ER marker ER-Tracker (C, D). Expression of PECP1-Ven (A, C) and PS2-Ven (B, D) with staining pattern of FM4-64 for plasma membrane marker (A, B) and ER-tracker (C, D) were merged. Bars = 10 μm.

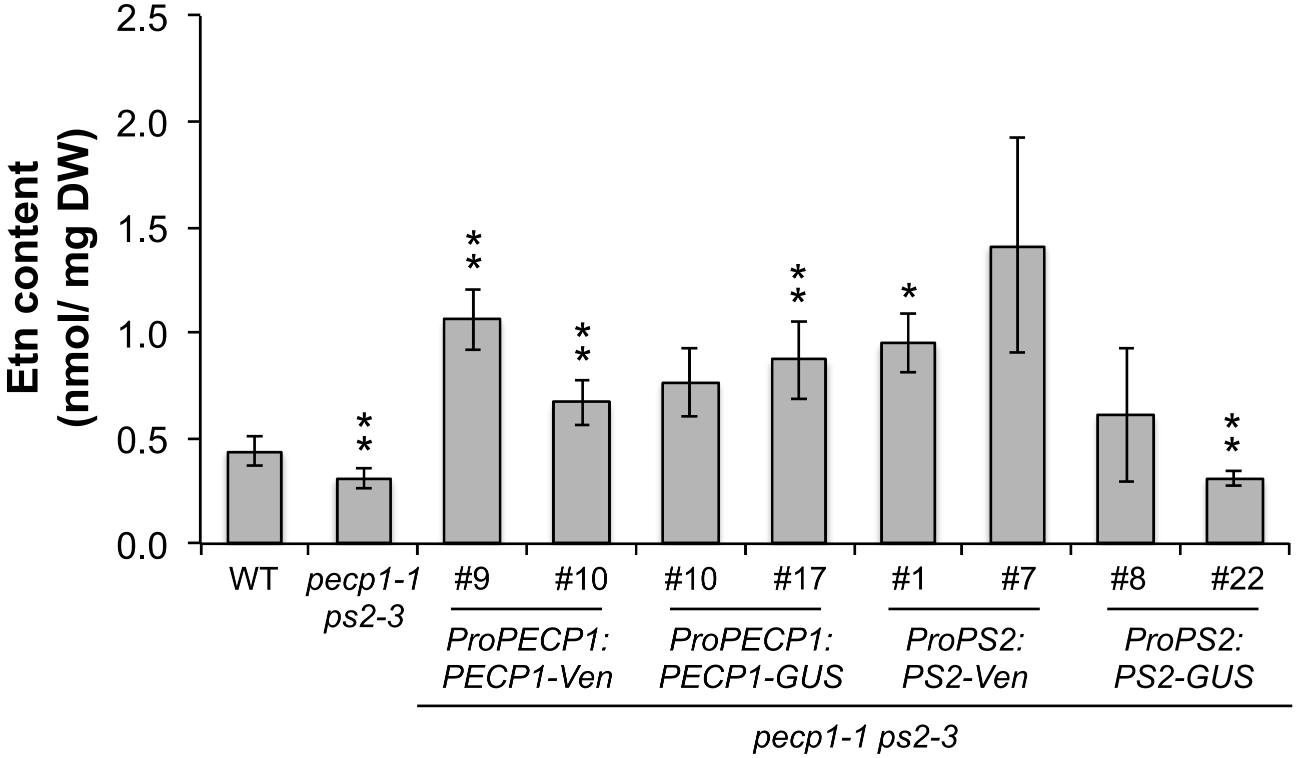

Supplementary Figure 4 | Transgenic plants rescued the reduced ethanolamine level of the double mutant *pepc1-1 ps2-3*. Ethanolamine content of phosphate-starved seedlings of the WT, *pepc1-1 ps2-3*, *ProPECP1:PECP1-Ven pecp1-1 ps2-3* (lines #9 and #10); *ProPECP1:PECP1-GUS pecp1-1 ps2-3* (lines #10 and #17); *ProPS2:PS2-Ven pecp1-1 ps2-3* (lines #1 and #7) and *ProPS2:PS2-GUS pecp1-1 ps2-3* (lines #8 and #22) cultured as described in the legend of Figure 5. Data are mean ± SD from 3 biological replicates. The asterisks indicate significance with reference to WT by Student's t-test (P<0.01, **; P<0.05, *).

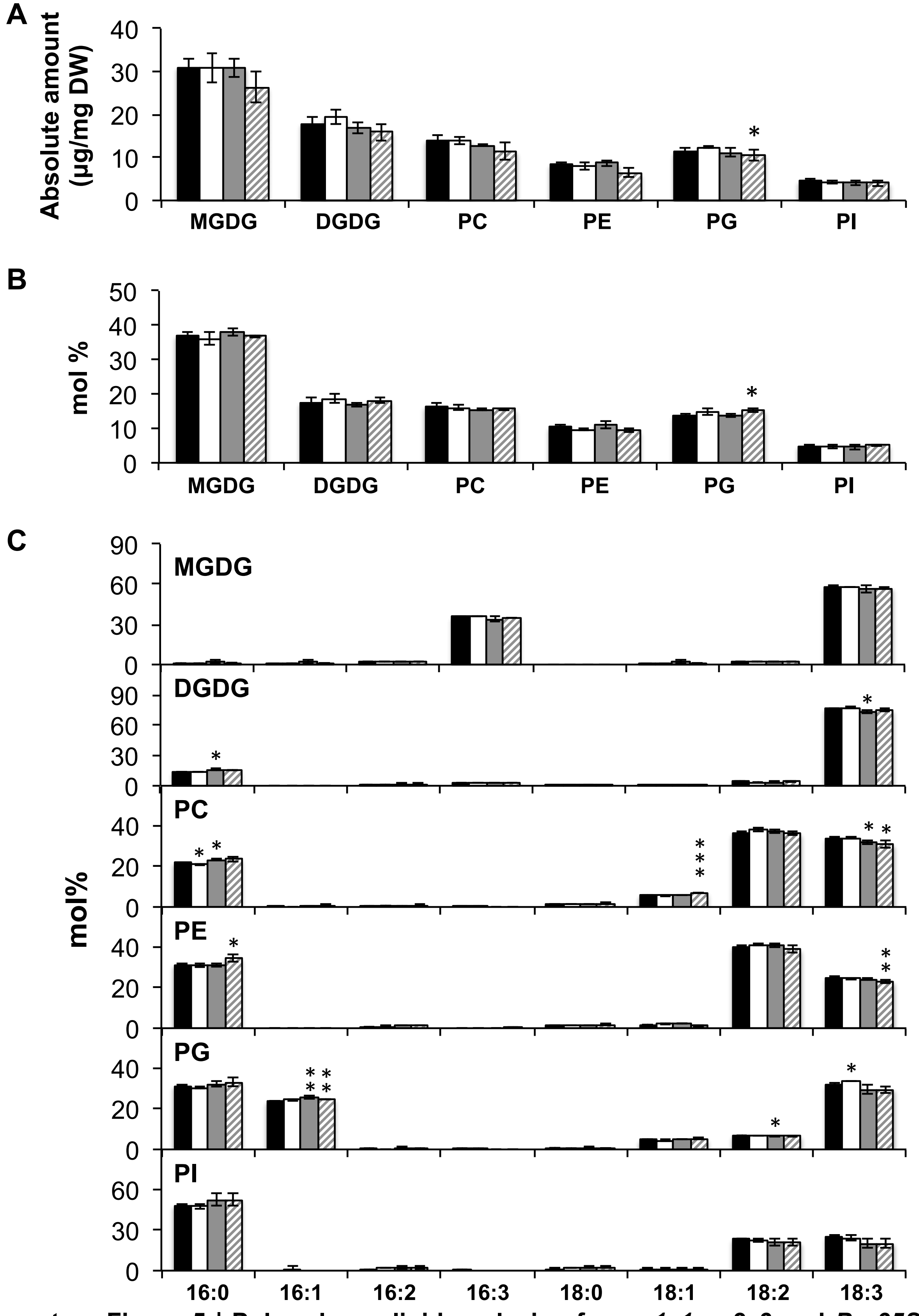

Supplementary Figure 5 | Polar glycerolipid analysis of *pecp1-1 ps2-3* and *Pro35S:PECP1* plants. (A,B) Contents of polar glycerolipid classes shown in absolute amount (µg lipid / mg dry weight) (A) and in mol% (B). (C) Fatty acid composition of each lipid classes. Total lipid was extracted from 20-day-old rosette leaves of WT (black bars), *pecp1-1 ps2-3* (white bars), and *Pro35S:PECP1* line #2 (gray bars) and line #5
(stripe bars), and lipid content was analyzed as described in Materials and Methods section. Data are mean ± SD from 3 biological replicates. Asterisks indicate statistical significance by Student's t-test (P<0.001, ***; P<0.01, **; P<0.05, *). MGDG, monogalactosyldiacylglycerol; DGDG, digalactosyldiacylglycerol; PC, phosphatidylcholine; PE, phosphatidylethanolamine; PG, phosphatidylglycerol; PI, phosphatidylinositol.

Supplementary Table 1. List of oligonucleotide primer sequences used in this study

No.	Sequence (5' to 3')
CH72	ATGGCCGCGGATATCACAAG
FG15	GGTTGCTCTCGTTGCCCTCCTAACATGTGCAAG
FG21	ACGCGTCGACATGGCTAAGAATAACAACATCGTGATCGTC
FG22	CGGAATTCTCACTTGACCAAATTTAAAGGAACTTGAATAGG TTCATGAACAATCCC
FG23	GATTTGATGGCTGGGATGACACGTGGATGAATTGGG
FG24	ACCAAATCTTCTCATGGTTGCT
FG25	CAGTAATCACCAGCACCATCTC
FG28	AGGATAGTGAGCGATGCAAACA
FG29	TGAAGTCGTGGTAGGGAGAGAT
KK97	CTGCAGGCGGCCGCACTAGTGATATC
KK98	CACTTCCTGATTATTGACCCACACTTTGCCG
KK104	GCGAAGCACTGCAGGCCGTAGCC
PK10	CGAATACACGTATCGTCATTGCAACACC
PK11	TGCTCTTCGTGTGTCTCAGTCTAGTGGCGCCTAGACGTTTTT GAAATAGTGGAGGA
PK13	GAGATGGAGCTGGCGATTACTGTCC
PK52	ACGCGTCGACATGGCTTACAATAGCAATAGCAATAACAACA ACAAC
PK54	CTAGTCTAGACTAACTAGACTGAGACACACGAAGAGCAC
PK66	CACCTGTTATGTCCAATGTTTATATTTCAGTGAATTCCACATG TC
PP68	TTCATTTGGAGAGGACAGCCCAAGCGTCGACTACGCGTCTC GAGATGAAGATCCCT
YN1397	CACCCTTACCTCAAGAAGAGTGTCGAGGGC
YN1398	GATACGAAAAATGAGCCCATTAAGCTTCC
YN1404	CAAGTTCCTTTAAATTTGGTCAAGGGCGCCTGATTGATGAA AACAAGAAATTGA
